# PD-1 and PD-L1 Checkpoint Signaling Inhibition for Cancer Immunotherapy: Mechanism, Combinations, and Clinical Outcome

**DOI:** 10.3389/fphar.2017.00561

**Published:** 2017-08-23

**Authors:** Hashem O. Alsaab, Samaresh Sau, Rami Alzhrani, Katyayani Tatiparti, Ketki Bhise, Sushil K. Kashaw, Arun K. Iyer

**Affiliations:** ^1^Use-Inspired Biomaterials and Integrated Nano Delivery Systems Laboratory, Department of Pharmaceutical Sciences, Eugene Applebaum College of Pharmacy and Health Sciences, Wayne State University Detroit, MI, United States; ^2^Department of Pharmaceutics and Pharmaceutical Technology, College of Pharmacy, Taif University Taif, Saudi Arabia; ^3^Department of Pharmaceutical Sciences, Dr. Harisingh Gour University Sagar, India; ^4^Molecular Therapeutics Program, Barbara Ann Karmanos Cancer Institute, Wayne State University, School of Medicine Detroit, MI, United States

**Keywords:** immunotherapy, PD-1/PDL-1 mechanism, tumor stroma role in PD-1/PD-L1, immune resistance, challenges and new approach, combination immune therapy, nivolumab, atezolizumab

## Abstract

Several cancers are highly refractory to conventional chemotherapy. The survival of tumors in several cases is assisted by checkpoint immunomodulation to maintain the imbalance between immune surveillance and cancer cell proliferation. Check point antibody inhibitors, such as anti-PD-1/PD-L1, are a novel class of inhibitors that function as a tumor suppressing factor via modulation of immune cell-tumor cell interaction. These checkpoint blockers are rapidly becoming a highly promising cancer therapeutic approach that yields remarkable antitumor responses with limited side effects. In recent times, more than four check point antibody inhibitors have been commercialized for targeting PD-1, PDL-1, and CTLA-4. Despite the huge success and efficacy of the anti-PD therapy response, it is limited to specific types of cancers, which attributes to the insufficient and heterogeneous expression of PD-1 in the tumor microenvironment. Herein, we review the current landscape of the PD-1/PD-L1 mechanistic role in tumor immune evasion and therapeutic outcome for cancer treatment. We also review the current progress in clinical trials, combination of drug therapy with immunotherapy, safety, and future of check point inhibitors for multiple types of cancer.

## Introduction

**Figure 1:**
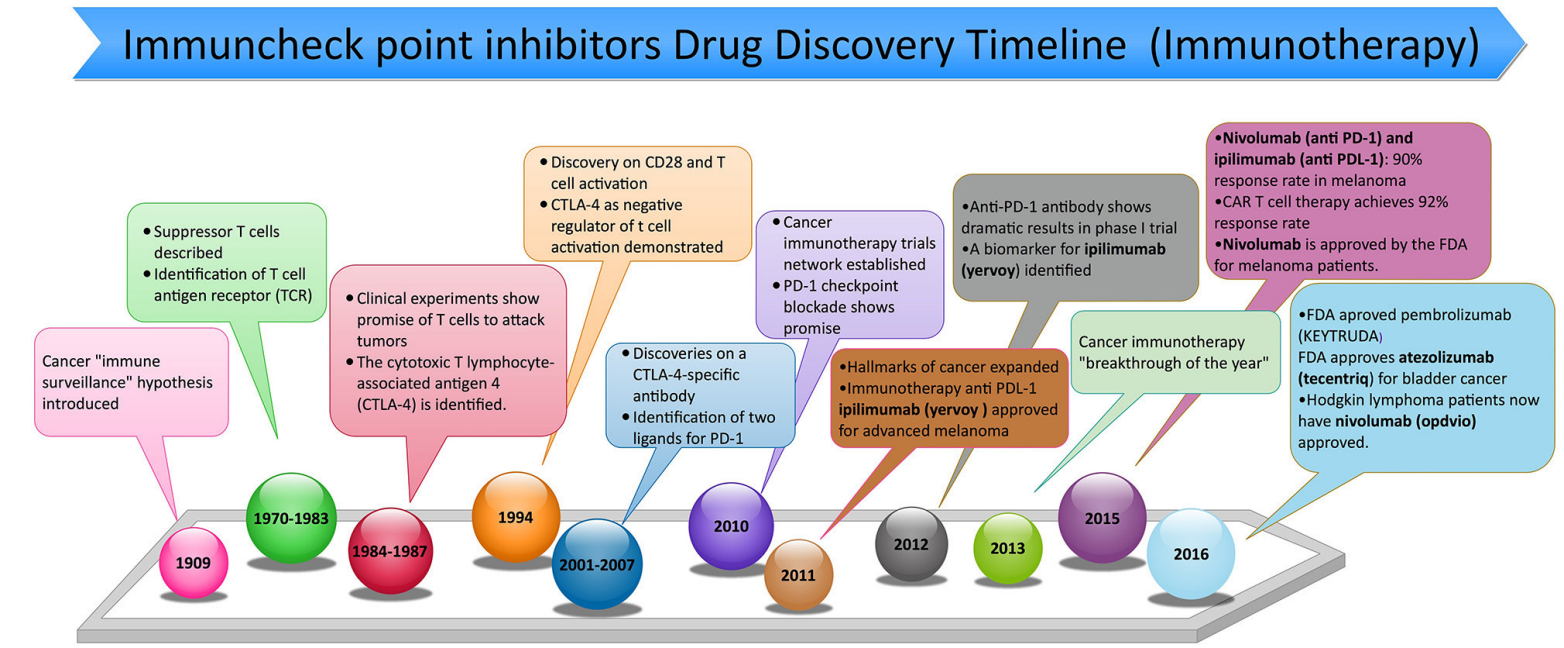
Timeline of discovery of anti-programmed death 1 (PD-1) and anti-programmed death ligand 1 (PD-L1) inhibitors used in cancer immunotherapy from ~1900s to February of 2017.

**Figure 2:**
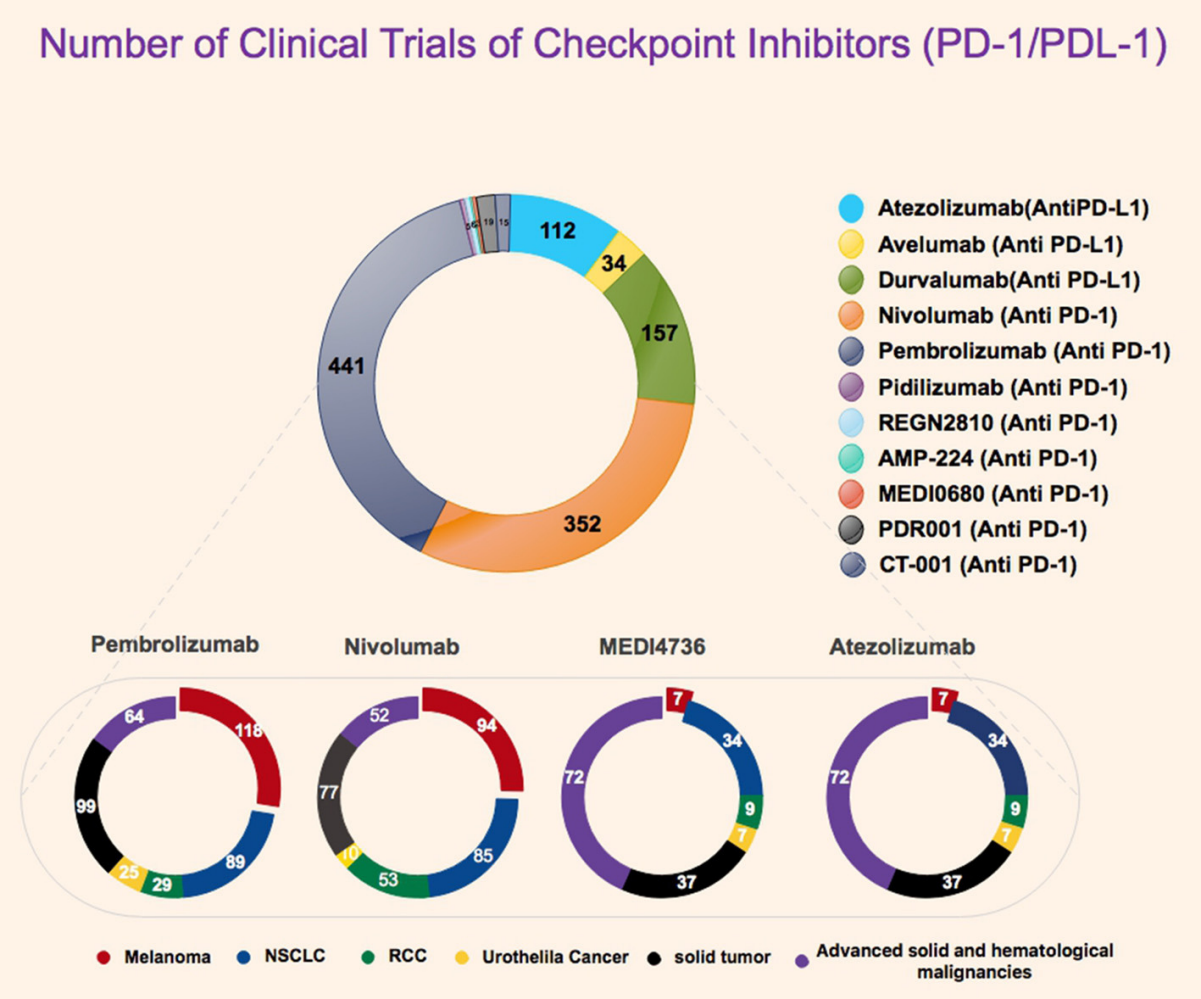
Statistics representative of number of clinical trials for PD-1 and PD-L1 inhibitor with highlight on currently for FDA approved PD-1/PDL-1 inhibitors. All data were obtained from FDA website, Clinicaltrials.gov, and National Cancer Institute.

Immunotherapy is an exciting approach, and tremendous strides have recently been made in our perception of the role of the host immune response in affecting tumor growth and response to various therapies (Pardoll, 2012). Through these advances, novel immune check point inhibitors have been identified and cleared for use in the clinic (Figure 1). The evolution of immune checkpoint inhibitors as anticancer treatment options represents one of the most successful approach in cancer drug discovery in the past few years (Couzin-Frankel, 2013). Indeed, immune checkpoint inhibitors have emerged as a frontline treatment for multiple cancers, such as metastatic melanoma, non-small cell lung cancer (NSCLC), renal cell carcinoma (RCCs), and bladder or urothelial cancer. They are presently being assessed in numerous other cancer types, including breast cancer, head and neck cancer, and some advanced solid and hematological malignancies. The loss of immunologic control has been confirmed as one of the emerging hallmarks of cancer (Hanahan and Weinberg, 2011) and in 1996, Leach et al. proposed an immune checkpoint blockade is an advanced strategy of cancer management (Leach et al., 1996). In 2011, the first immune checkpoint inhibitor (ipilimumab as an anti-CTLA-4 antibody) was approved by the FDA as for the treatment of melanoma that crated a footstep in immunotherapy cancer treatment (Lipson and Drake, 2011; Sondak et al., 2011; Barbee et al., 2015). Currently, the two classes of immunotherapy that have been FDA approved for clinical use are (1) inhibitors of either the programmed death receptor 1 (PD-1) or its ligand (PD-L1), or (2) cytotoxic T-cell lymphocyte-associated protein 4 (CTLA-4) (Michot et al., 2016). New agents that target other aspects of the immune system are currently under development (Figure 2). T cells mediated cellular immunity is firmly managed and controlled by a check/balance system that functions due to many stimulatory and inhibitory proteins. The inhibitory receptors, also called immune checkpoints, regulate CTL activation and effector functions to sustain self-tolerance and minimize bystander tissue damage as an eventual result of immune response vs. pathogenic invasion (Pardoll, 2012). Targeting both PD-1 and PD-L1, the immune checkpoint inhibitors agents could reactivate cytotoxic T cells to work against cancer cells. When the T cell receptor (TCR) of a T cell distinguishes antigens in the presence of major histocompatibility complex (MHC), the immune checkpoint molecule modulates signaling of co-stimulatory factors such as CD28 to amplify the signal, whereas co-inhibitory molecules suppress it. Recent advancement has indicated that the expression of immune-inhibitory checkpoints such as PD-1/PD-L1 and CTLA-4 function as potent mediators for the balance and escape phases of cancer immune editing. These molecules are expressed on activated T cells, but when they adhere to ligands either on APC (CTLA-4 binding to CD80/CD86) or tumor cells (PD-1 binding to PD-L1), they tend to suppress the antitumor response. Efforts to use monoclonal antibodies (mAbs) to target and block these immunoinhibitory interactions have led to a new era of immunotherapy based agents for cancer therapy (Freeman et al., 2000; Topalian et al., 2012; Ott et al., 2013).

New immunotherapies for treating cancer focus on moving the balance from a pro-tumor to an antitumor microenvironment; as a result, this allows the immune system to maximize an efficient antitumor response. Also, negative regulatory pathways are key targets. The anti–CTLA-4 monoclonal antibody (mAb) ipilimumab improved the survivability of patients with metastatic melanoma undergoing phase 3 trials (Hodi et al., 2010). Ipilimumab was subsequently approved by the United States Food and Drug Administration (USFDA) for patients with advanced melanoma. A previous report on an early-stage trial granted preliminary activity data of ipilimumab in patients with castrate-resistant prostate cancer (CRPC) (Slovin et al., 2013). The fully human anti-PD-1 mAb BMS-936558 (Nivolumab), investigated in renal cell cancer (RCC), melanoma, non-small cell lung carcinoma (NSCLC), and colorectal cancer (CRC), demonstrated antitumor activity in phase 1/1b studies before its eventual FDA approval for multiple cancers (Gettinger et al., 2014; Topalian et al., 2014). The humanized anti-PD-1 antibody MK-3945 (Pembrolizumab) has also showed an anticancer response in patients with some solid cancers in a multiple phases clinical trials before its FDA approval for multiple cancers (Garon et al., 2015; Robert et al., 2015; Herbst et al., 2016). Also, CT-011 (Pidilizumab), a humanized anti-PD-1 antibody, has been tested in various hematologic malignancies, showing potential clinical impact in patients with non-Hodgkin's lymphoma, chronic lymphocytic leukemia, Hodgkin's lymphoma, multiple myeloma, and acute myeloid leukemia (Berger et al., 2008; Atkins et al., 2014; Westin et al., 2014). Finally, the anti-PD ligand 1 (PD-L1) mAb Atezolizumab and Durvalumab showed preliminary antitumor activity in multiple solid cancers before their recent FDA approval for multiple cancers (Rizvi et al., 2015; Antonia et al., 2016; Fehrenbacher et al., 2016; McDermott et al., 2016; Rosenberg et al., 2016).

This review introduces the PD-1 and PD-L1 signaling pathways as well as the negative regulatory pathway in antitumor immune responses. The review also focuses on the pre-clinical trials, clinical trial development, and research advancement of anti-PD-1 and anti-PD-L1 mAbs for treating variety of cancers. Additionally, recent attempts to advance combination regimens using PD-1 and PD-L1 blockade as a standard therapy are described.

## Programmed death-1 (PD-1) and its ligand PD-L1

Recent research has granted a clearer perceptive on the factors that reduce an antitumor immune response, leading to the discovery of several agents that work on immune costimulatory and inhibitory checkpoint pathways. A good example of the advanced checkpoint molecules that mediates tumor-induced immune suppression is programmed death-1 (PD-1). Physiologically, the PD-1/PD-L1 pathway has emerged as a result of the need to control the degree of inflammation at locations expressing the antigen, in order to secure normal tissue from damage. There is a remarkable expression of the PD-1 protein on the surface of all activated T cells. When a T cell recognizes the antigen expressed by the MHC complex on the target cell, inflammatory cytokines are produced, initiating the inflammatory process. These cytokines result in PD-L1 expression in the tissue, activating the PD-1 protein on the T cell leading to immune tolerance, a phenomenon where the immune system lose the control to mount an inflammatory response, even in the presence of actionable antigens (Mahoney et al., 2015). In certain tumors, most remarkably in melanomas, this protective mechanism is perverted through overexpression of PD-L1; as a result, it circumvents the generation of an immune response to the tumor. PD-1/PD-L1 inhibitors pharmacologically prevent the PD-1/PD-L1 interaction, thus facilitating a positive immune response to kill the tumor. Although, PD-1/PD-L1 inhibitors have clear and demonstrative benefits as anticancer agents, one limitation to their use is that their activity is dependent on the generation of a population of T cells capable of recognizing the tumor through antigen-presenting cells (APCs). If this process does occur, blocking PD-1/PD-L1 is ineffecient, as there is a lack of immune response to unleash the effective killing of the tumor cells. While tumor expression of PD-L1 might be suggestive of a tumor that is subduing an immune response and as a result could serve as a potential biomarker for clinical benefit, it is clear that not all PD-L1-expressing tumors respond to PD-1/PD-L1 inhibitors. Conversely, it has been noted that PD-L1-negative tumors can respond to these agents (Aguiar et al., 2016). Further work on this question is ongoing.

## Mechanism of PD-1/PD-L1 signaling

**Figure 3:**
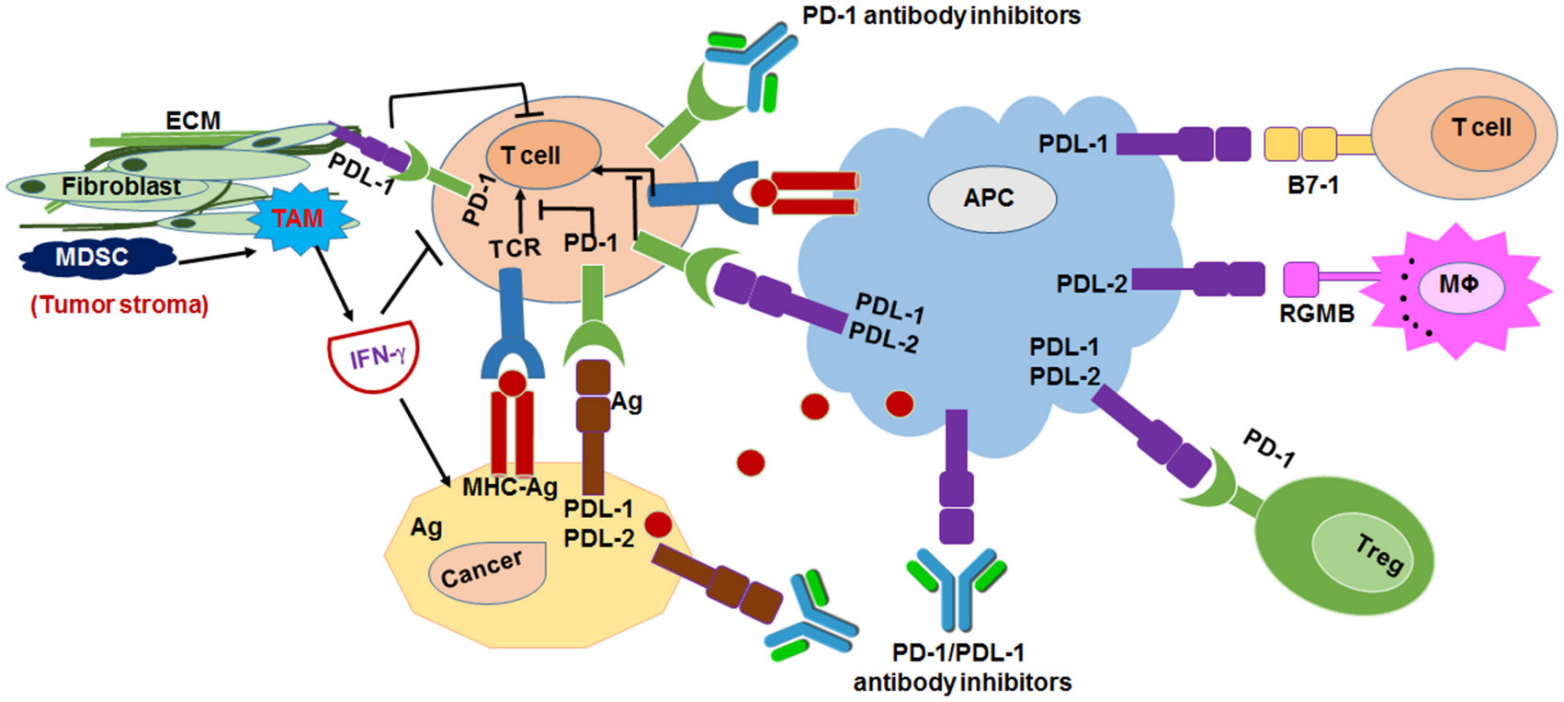
Mechanism of anti-programmed death 1 (PD-1) receptor and anti-programmed death ligand 1 (PD-L1)/L2 inhibitors mediated cancer immunotherapy. Antigen-presenting cells (APCs) bind to antigen (Ag) that released from tumor cells and T cells to activate T-cell receptor (TCR) and MHC binding. PD-L1 of tumor stroma interacts with PD-1 of T cells to suppress the T-cell mediated tumor cytotoxicity. Tumor associated macrophage (TAM), myeloid derived suppressor cells (MDSC) has crucial role in PD-1/PD-L1 mediated tumor immunosuppression (Ohaegbulam et al., 2015).

In the tumor microenvironment, PD-1 and its ligand PD-L1 perform a vital role in tumor progression and survival by escaping tumor neutralizing immune surveillance. It has been found that PD-1 is expressed on a variety of immune cells, such as monocytes, T cells, B cells, dendritic cells, and tumor-infiltrating lymphocytes (TILs). However, PDL-1 is expressed in tumor cells and antigen presenting cells (APCs), and the engagement of PD-L1 with PD-1 of T cell creates T cell dysfunction, exhaustion, neutralization, and interleukin-10 (IL-10) production in a tumor mass (Sun et al., 2015). Therefore, the function of a tumor overexpressing PD-L1 is to protect itself from cytotoxic T cell (CD8+) mediated cell killing (Zou and Chen, 2008). Another interacting molecule such as B7-1 (CD80), a protein expressed on activated T cells and APCs, interacts with the PD-L1 of tumor cells causing the negative regulation of effector T-cell activation (Butte et al., 2009). Due to exhaustion of CD8+ T cells, tumor cells become very aggressive and secrete several pro-inflammatory cytokines, such as tumor necrosis factor alpha (TNF-α), interleukin-2 (IL-2), and interferon gamma (IFN-γ). Another subtype of T cells, such as regulatory T cells (Treg, CD4+ Foxp3+) create a highly immunosuppressive tumor environment through maintaining the expression of PD-1 on its surface (Francisco et al., 2010). It has been found that in the presence of CD3 and TGF-β, the PD-1 receptor of Treg cells increases the *de novo* transformation of naive CD4+ T cells to Treg cells, thus attenuating immune responses. This conversion increases Treg expression and immune suppressive function of CD4+ T-cell through inhibition of mammalian target of rapamycin (mTOR)-Akt signaling cascade (Ohaegbulam et al., 2015). Thus, the presence of PD-1 expression not only suppresses effector T-cell function but also increases the conversion of the immunosuppressive Treg cell population. Even though PD-1 has widely been studied in T-cells, its functions in B-cells have also become apparent for tumor immunosuppression. It has been found that PD-1 expression is highly regulated during B cell differentiation, but PD-1 levels are insignificant in pro-B cells (an early stage of mature B cell) and increase with B cell differentiation (Thibult et al., 2013). Additionally, maturation of B-cells can significantly be enhanced by PD-1 activated toll-like receptor 9 (TLR9) agonists. Thus, inhibition of PD-1 function on B cells has been shown to enhance antigen-specific antibody responses, indicating that PD-1 plays a role in suppressing B cell mediated T-cell activation (Ohaegbulam et al., 2015). PD-1 has two binding partners, namely, PD-L1 (B7-H1) and PD-L2 (B7-DC), and among them, PD-L1 is responsible for tumor immune modulation. The binding affinity of PD-1 with PD-L1 is three times greater than the affinity between PD-1 for PD-L2. PDL-1 expressions in tumor cells and hematopoietic cells are determined by the stimulation of pro-inflammatory cytokines such as IFN-γ and TNF-α. Although, PD-L1 is expressed in a wide range of hematopoietic and non-hematopoietic cells, PD-L2 has restricted expression on macrophages, dendritic cells (DCs) and mast cells in the secretion of IL-4 and IFN–γ. It has been recently reported that PD-L2 interacts with repulsive guidance molecule B (RGMB) of macrophage (MΦ) proteins (Xiao et al., 2014; Figure 3). Although, there are several reports on PD-L2, little information is available about its role in cancer immunosuppression. Another important check point molecule, CTLA4 is broadly engaged in tumor immune evasion through the down-regulation of CD4+ T effector (Teff) cells and the enhancement of Treg cell activity (Topalian et al., 2016). CTLA-4, a homolog of CD28 (a costimulatory factor of the T-cell receptor), binds to the B7-1/2 protein of APCs and determines whether T cells will undergo activation or suppression. It is believed that CTLA-4 binding to B7 yields T-cell inhibitory signals, which also depends on stimulation T-cell receptors (TCR) and MHC-Ag binding (Topalian et al., 2016). Therefore, the combined inhibition of the PD-1/PD-L1, and CTLA-4/B7 axis has been established to be an effective anti-tumor treatment strategy for patients with various malignancies (Postow et al., 2015). PD-L1 of tumor stromal components, such as fibroblast, extracellular matrix (ECM), tumor associated macrophages (TAM), and myeloid derived suppressor cells (MDSC) deactivates T-cell (CD8+) mediated cancer cells killing through interaction with PD-1 In review on T-cell surface (Sznol and Chen, 2013; Turley et al., 2015). Similarly, maturation of MDSC to TAM and secretion of pro-inflammatory cytokines (IFN-γ) from TAM suppress T-cell functions, thus providing positive modulation of PD-1 and PD-L1 interaction (Turley et al., 2015).

## Mechanism of PD-1/PD-L1 mediated immune resistance

**Figure 4:**
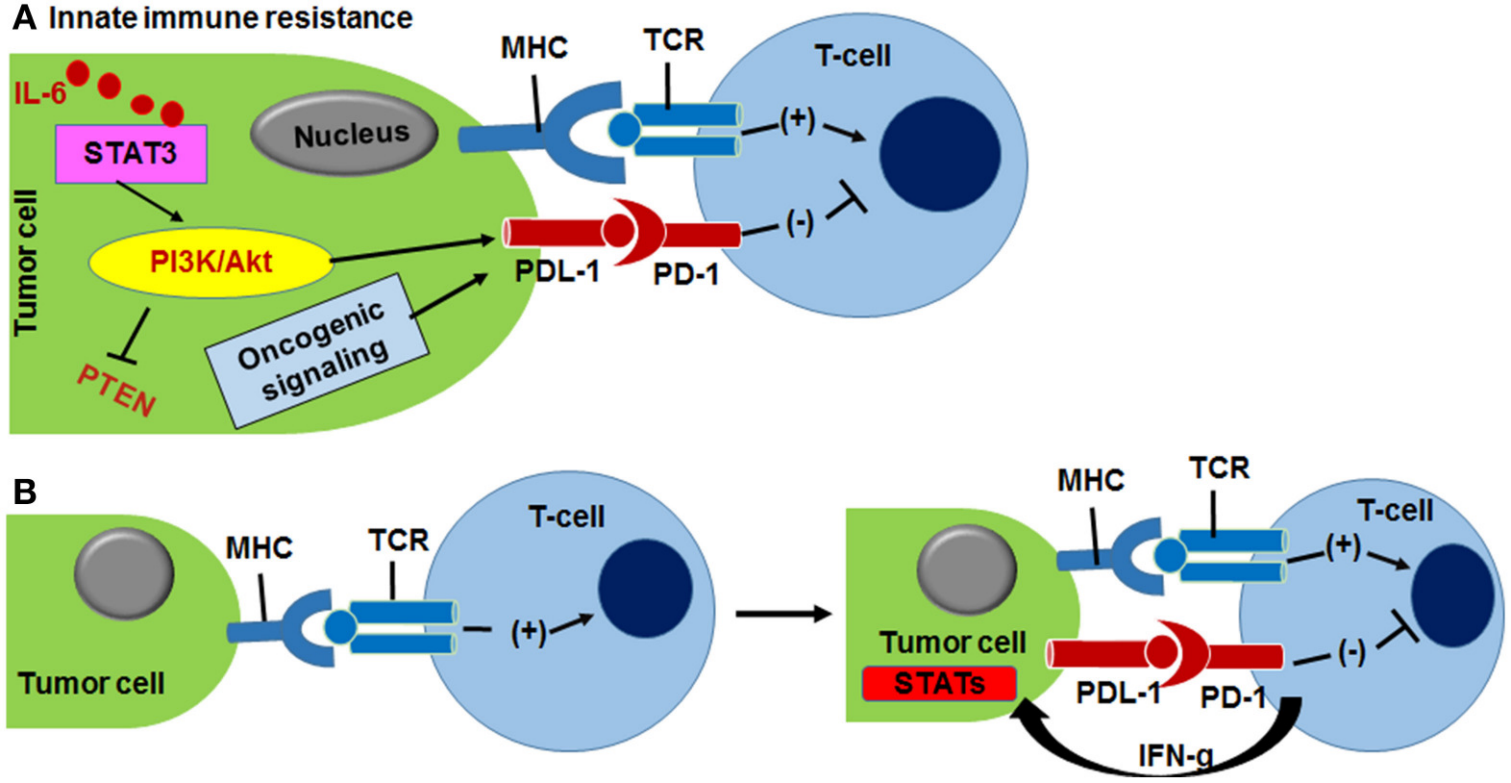
**(A)** Innate immune resistance is driven by activation of PI3K/Akt kinase and IL-6/STAT3 oncogenic signaling that up-modulate PDL-1 protein expression in tumor cells, resulting PD-1/PD-L1 complexation. **(B)** Adaptive immune resistance of cancer cells is outcome of INF-γ responded PDL1 expression. MHC and TCR interaction helps T-cell activation (Pardoll, 2012).

PD-1 associated immune-resistance depends on the accessibility of PD-L1 ligand in the tumor. The PD-L1 expression is monitored either by upregulation of PI3K-Akt kinases or secretion of IFN-γ, and due to PD-L1 expression, variability in two general types of immune resistance is observed, namely, (I) innate immune resistance, and (II) adaptive immune resistance (not to be confused with innate and adaptive immunity; Pardoll, 2012; Figure 4). With innate immune resistance, in glioblastomas the PD-L1 expression is driven by downregulation of PTEN which is linked to activation of PI3K-Akt tumorigenic signaling (Pardoll, 2012). Similarly, the unresponsiveness of PD-1 blockade therapy in prostate cancers has been attributed to the PD-L1 mediated innate immune resistance (Martin et al., 2015). Certain lymphomas and lung cancers have been reported to drive PD-L1 expression through the upregulation of the signal transducer and activator of transcription 3 (STAT3) and lymphoma kinase (ALK) signaling resistance (Pardoll, 2012). The STAT3 activation is modulated through pro-inflammatory cytokines, such as IL-6 and the IL-6-STAT3 axis is considered as one of the crucial pathway in tumorigenic macrophage polarization and immune suppression (Sau and Banerjee, 2014; Sau et al., 2017). In adaptive immune resistance, in some tumors, the PD-L1 expression is induced due to the secretion of pro-inflammatory IFN-γ from tumor and tumor-stromal cells that neutralize CD8+ cytotoxic T cell induced anti-tumor immune responses. The adaptive immune response in various preclinical and clinical studies represents an alternative mechanism of conventional drug resistance that involves the mutation of the drug targets (Ribas, 2015).

## PD-1/PD-L1 inhibition and their inhibitors

The presence of PD-1 and PD-L1 has a major role in the inhibition of effector T-cell function (Pardoll, 2012). Clinical studies have indicated that antibodies blocking PD-1 and PD-L1 have a reliable effect on many advanced malignancies (Agata et al., 1996). PD-1 and PD-L1 targeting is an efficient way to maintain the function of effector T-cells. Monoclonal antibodies (mAbs) are a class of drugs called checkpoint inhibitors that inhibit the interaction of PD-1 and PD-L1 and overcome the disadvantages of conventional anticancer therapy. *In vitro* and *in vivo* studies that were done by Lussier et al. showed that blocking PD-1 using an antibody can partially enhance T-cell function (Lussier et al., 2015). MAbs can significantly reduce toxicity well within tolerable limits, while being able to shrink solid tumors, suppress advanced tumors and metastasis, and overall improve patient survival (Topalian et al., 2014; Naidoo et al., 2015). Hundreds of clinical trials on anti-PD-1 and PD-L1 mAbs are under active development. Some of them having entered phase 3 clinical trials and are benefiting many patients. The FDA has recently granted approval to some anti-PD-1and PD-L1 mAbs targeting a range of human cancers. The clinical activity of anti-PD-1and PD-L1 mAbs holds promise in targeting PD-1 and PD-L1 immune checkpoints, thereby ameliorating patient conditions significantly (Chen and Han, 2015). The anti-PD-1 therapies approved by the FDA and under active clinical trials for renal cell carcinoma, NSCLC (non-small cell lung carcinoma), HNSCC (head and neck squamous cell carcinoma), and bladder (urothelial) cancer are summarized below and noted in Tables 1, 2. The PD-1 and PD-L1 is a receptor-ligand system and in tumor microenvironment they are attached to each other, resulting blockade of anti-tumor immune responses. PD-1 is majorly expressed on the T cells of the immune system, whereas PD-L1 is on the cancer cells and antigen- presenting cells. Therefore, the inhibitors that block the interaction of PD-1 and PD-L1 will cause resurrection of T-cell mediated anti-tumor immune effect. The PD-1 and PD-L1 antibody inhibitors have been designed to block either the PD-1 or the PD-L1 side and turn on T-cell mediated immunity. Currently, it is not clear whether the PD-1 and PD-L1 inhibitors are more effective. The effectiveness of PD-1 and PD-L1 inhibitors depends on patients' characteristics, such as (i) gender, (ii) types of tumors, (iii) mutation, translocation of genes (EGFR, Kras, ALK), and (iv) metastases of tumor (D'Incecco et al., 2015). As tumor is heterogeneous in nature, the expression of PD-L1 is not uniform, thus PD-L1 immunohistochemistry staining varies with tumor locations. Therefore, indication of PD-L1 expression and response of PD-L1 inhibitors remain debatable and needs to be understood deeply (Wang et al., 2016). Similarly, PD-1 expression also depends of tumor patient's characteristics Immune response: The phase I studies with anti-PD-1 drugs, such as Nivolumab and Pembrolizumab with non-small-cell lung cancer, advanced melanoma, renal cell carcinoma, and other solid tumor patients have demonstrated very promising response with minimal side effects. Inspired from phase I response, PD-1 blockers were studied for further trials and in phase III trial patients with advance melanoma showed excellent response than the NSCLC, RCC. For this reason, Nivolumab received FDA approval for the first line treatment for advanced melanoma, and second line treatment for squamous NSCLC, RCC. Similarly, Pembrolizumab is the first line treatment option for metastatic melanoma, metastatic non-small cell lung cancer (NSCLC; Postow et al., 2015). Anti-PD-L1, such as Atezolizumab is the first FDA approved PD-L1 blocker, and has been used as first line treatment for cisplatin resistant metastatic urothelial carcinoma, metastatic NSCLC. Variety of clinical trials are under progress for colorectal cancer, bladder cancer, renal cell carcinoma, head and neck cancer, and GI malignancies.

**Table 1:** Some selected immunotherapeutic agents (Anti- PDL-1) in clinical trials including the possible combination therapy.

**CT Number**	**Status**	**Phase**	**Condition**	**Sponsor**	**Additional agents**
**ATEZOLIZUMAB (PDL-1 INHIBITOR)- APPROVED BY FDA**					
NCT02724878	Recruiting	II	Non Clear Cell Kidney Cancer	Dana-Farber Cancer Institute	Bevacizumab
NCT02989584	Recruiting	I, II	Bladder Cancer, Metastatic Bladder Cancer, Urothelial Carcinoma	Memorial Sloan Kettering Cancer Center	Gemcitabine
					Cisplatin
NCT02302807	Not yet recruiting	III	Bladder Cancer	Hoffmann-La Roche	Docetaxel
					Paclitaxel
					Vinflunine
NCT02708680	Recruiting	I, II	Breast Cancer	Syndax Pharmaceuticals	Entinostat
NCT03023423	Recruiting	I, II	Non-Small-Cell Lung Carcinoma	Janssen Research & Development, LLC	Daratumumab
NCT02716038	Recruiting	II	Carcinoma, Non-Small-Cell Lung	Columbia University, Genentech, Inc., Celgene Corporation	MPDL3280A
					Carboplatin
					Nab-paclitaxel
NCT02814669	Recruiting	I	Castrate-Resistant Prostate Cancer	Hoffmann-La Roche	Radium-223 dichloride
NCT02846623	Recruiting	II	Chronic Lymphocytic Leukemia	M.D. Anderson Cancer Center	Obinutuzumab
			Small Lymphocytic Lymphoma		
NCT02788279	Recruiting	III	Colorectal Cancer	Hoffmann-La Roche	Cobimetinib
					Regorafenib
NCT02792192	Recruiting	I, II	High-risk Non-muscle-invasive Bladder Cancer (NMIBC)	Hoffmann-La Roche	Biological: Bacille Calmette-Guérin
NCT02902029	Recruiting	II	Malignant Melanoma	University Hospital, Essen	Vemurafenib
					Cobimetinib
NCT02908672	Recruiting	III	Melanoma	Hoffmann-La Roche	Vemurafenib
NCT02924883	Recruiting	II	Metastatic Breast Cancer	Hoffmann-La Roche	Trastuzumab
NCT02425891	Recruiting	III	Metastatic Breast Cancer; TNBC	Hoffmann-La Roche	Nab-Paclitaxel
NCT03024437	Not yet recruiting	I, II	Metastatic Cancer	Roberto Pili	Bevacizumab
					Entinostat
				Renal Cancer
NCT03013218	Not yet recruiting	II	Metastatic Cancer; Solid Tumor; Advanced Cancer; Non-Hodgkin Lymphoma	Alexo Therapeutics, Inc.	Trastuzumab
NCT02657434	Recruiting	III	NSCLC	Hoffmann-La Roche	Carboplatin
					Pemetrexed
					Cisplatin
NCT01903993	Active-not recruiting	II	NSCLC	Hoffmann-La Roche	Docetaxel
NCT02008227	Active, not recruiting	III	NSCLC	Hoffmann-La Roche	Docetaxel
NCT02367781	Recruiting	III	Non-Squamous Non-Small Cell Lung Cancer	Hoffmann-La Roche	Carboplatin
					Nab-paclitaxel
**ATEZOLIZUMAB (PDL-1 INHIBITOR)- APPROVED BY FDA**					
NCT02366143	Recruiting	III	NSCLC	Hoffmann-La Roche	Bevacizumab
					Carboplatin
					Paclitaxel
NCT02891824	Recruiting	III	Ovarian Cancer	ARCAGY/GINECO GROUP	Avastin + platinum-based chemotherapy
NCT03038100	Not yet recruiting	III	Ovarian Cancer; Fallopian Tube Cancer; Peritoneal Neoplasms	Hoffmann-La Roche	Paclitaxel
					Carboplatin
					Bevacizumab
NCT02659384	Recruiting	II	Ovarian Neoplasms	EORTC	Bevacizumab acetylsalicylic acid
NCT02992912	Recruiting	II	Patients with Metastatic Tumors	Gustave Roussy, Cancer Campus, Grand Paris	SABR
NCT03016312	Recruiting	III	Prostatic Neoplasms	Hoffmann-La Roche	Enzalutamide
			Castration-Resistant		
NCT02873195	Not yet recruiting	II	Recurrent Colorectal Carcinoma; Stage IVA Colorectal Cancer; Stage IVB Colorectal Cancer	Academic and Community Cancer Research, (NCI)	Bevacizumab
					Capecitabine
NCT02926833	Recruiting	II	Refractory Diffuse Large B Cell Lymphoma	Kite Pharma, Inc. Genentech, Inc.	Biological: KTE-C19
NCT02420821	Recruiting	III	Renal Cell Carcinoma	Hoffmann-La Roche	Bevacizumab
					Sunitinib
NCT02748889	Recruiting	II	Small Cell Lung Cancer (SCLC)	Giuseppe Giaccone,Vanderbilt University, |Georgetown University	Etoposide MPDL3280A
NCT02763579	Recruiting	III	Small Cell Lung Cancer	Hoffmann-La Roche	Carboplatin
					Etoposide
NCT02367794	Recruiting	III	Squamous NSCLC	Hoffmann-La Roche	Carboplatin
					Nab-paclitaxel
NCT02409355	Active, not recruiting	III	Squamous NSCLC	Hoffmann-La Roche	Carboplatin
					Cisplatin
NCT02807636	Recruiting	III	Urothelial Carcinoma	Hoffmann-La Roche	Carboplatin
					Gemcitabine
					Cisplatin
NCT03029832	Not yet recruiting	II	Urothelial Carcinoma	Genentech, Inc.	MOXR0916
**AVELUMAB (ANTI-PDL-1) APPROVED BY FDA**					
NCT02875613	Recruiting	II	Nasopharyngeal Cancer	Assuntina Sacco, M.D., Pfizer, University of California, San Diego	–
NCT02912572	Not yet recruiting	II	Metastatic Endometrial Cancer	Dana-Farber Cancer Institute, Pfizer	–
NCT02915523	Not yet recruiting	I, II	Epithelial Ovarian Cancer; Peritoneal Cancer; Fallopian Tube Cancer	Syndax Pharmaceuticals Merck KGaA, Pfizer	Entinostat
NCT02953561	Not yet recruiting	I, II	Relapsed Acute Myeloid Leukemia; Refractory Acute Myeloid Leukemia; Acute Myeloid Leukemia	M.D. Anderson Cancer Center, Pfizer	5-azacytidine
NCT02943317	Recruiting	II	Epithelial Ovarian Cancer	Verastem, Inc.	VS-6063
NCT02994953	Recruiting	II	Advanced Solid Tumors	Merck KGaA, EMD Serono	NHS-IL12
NCT02952586	Recruiting	III	Squamous Cell Carcinoma of the Head and Neck	Pfizer	Chemo-radiation
NCT02395172	Recruiting	III	NSCLC	Merck KGaA, EMD Serono	Docetaxel
NCT02968940	Not yet recruiting	II	Glioblastoma	New York University School of Medicine, EMD Serono	Radiation: (HFRT)
NCT02580058	Recruiting	III	Ovarian Cancer	Pfizer	Biological: PLD
NCT02684006	Recruiting	III	Renal Cell Cancer	Pfizer	Axitinib
					Sunitinib
NCT03035630	Not yet recruiting	II	Clear-cell Renal Cell Carcinoma;	Guru Sonpavde, Hoosier Cancer Research Network, Pfizer	Sunitinib
NCT02625623	Recruiting	III	Unresectable, Recurrent, Locally Advanced or Metastatic Gastric or Gastroesophageal Junction Adenocarcinoma, Gastric Cancer Third Line	Merck KGaA,EMD Serono	Irinotecan
					Paclitaxel
NCT02576574	Recruiting	III	First Line NSCLC	Merck KGaA, EMD Serono	Pemetrexed
					Paclitaxel
					Gemcitabine
					Carboplatin
					Cisplatin
					Carboplatin
NCT02603432	Recruiting	III	Urothelial Cancer	Pfizer	–
NCT02625610	Recruiting	III	Unresectable, Locally Advanced or Metastatic, Adenocarcinoma of the Stomach, or of the Gastro Esophageal Junction	Merck KGaA, EMD Serono	Oxaliplatin
					5-Fluorouracil
					Leucovorin
					Capecitabine
					PD 0360324
NCT02718417	Recruiting	III	Ovarian Cancer	Pfizer	Carboplatin paclitaxel
NCT02584634	Recruiting	II	NSCLC	Pfizer	|PF06463922 Crizotinib
NCT02951156	Not yet recruiting	III	Diffuse Large B-Cell Lymphoma (DLBCL)	Pfizer, EMD Serono	Utomilumab
					Rituximab
					Azacitidine
					Bendamustine
					Gemcitabine
					Oxaliplatin

*Data taken from Clinicaltrials.gov website*.

**Table 2:** Some selected immunotherapeutic agents (Anti- PD-1) in clinical trials including the possible combination therapy.

**CT Number**	**Status**	**Phase**	**Condition**	**Sponsor**	**Additional agents**
**PIDILIZUMAB (CT001) (ANTI-PD-1)**					
NCT02530125	Active, not recruiting	II	Stage III Diffuse Large B-Cell Lymphoma; Stage IV Diffuse Large B-Cell Lymphoma	Northwestern University; Gateway for Cancer Research; National Cancer Institute (NCI)	–
NCT02077959	Active, not recruiting	I/II	Multiple Myeloma	Yvonne Efebera; CureTech Ltd; Ohio St. Univ. Comprehensive Cancer Center	lenalidomide
NCT00532259	Completed	III	Lymphoma, Large Cell, Diffuse; Lymphoma, Mixed Cell, Diffuse; Primary Mediastinal Large B-Cell Lymphoma	Cure Tech Ltd	–
NCT01435369	Completed	II	Melanoma; Malignant Melanoma	Medivation, Inc.	–
NCT00532259	Completed	II	Lymphoma, Large Cell, Diffuse; Lymphoma, Mixed Cell, Diffuse; Primary Mediastinal Large B-Cell Lymphoma	CureTech Ltd	–
NCT00890305	Completed	II	Metastatic Colorectal Cancer	Medivation, Inc.	FOLFOX
NCT02077959	Active, not recruiting	II	Multiple Myeloma	Yvonne Efebera; CureTech Ltd; Ohio State University Comprehensive Cancer Center	Lenalidomide, pidilizumab
NCT02530125	Active, not recruiting	II	Stage III Diffuse Large B-Cell Lymphoma; Stage IV Diffuse Large B-Cell Lymphoma	Northwestern University; Gateway for Cancer Research; National Cancer Institute (NCI)	Pidilizumab
**REGN2810 (ANTI-PD-1)**					
NCT03002376	Not yet recruiting	II	Melanoma	Regeneron Pharmaceuticals; Sanofi	–
NCT02760498	Recruiting	II	Advanced Cutaneous Squamous Cell Carcinoma	Regeneron Pharmaceuticals	–
**AMP-224 (ANTI-PD-1)**					
NCT02298946	Active, not recruiting	I	Colorectal Cancer; Colorectal Neoplasms; Colorectal Carcinoma	National Cancer Institute (NCI); National Institutes of Health Clinical Center (CC)	Cyclophosphamide
NCT01352884	Completed	I	Cancer	MedImmune LLC; GlaxoSmithKline	–
**MEDI0680 (ANTI-PD-1)**					
NCT02118337	Recruiting	I	Select Advanced Malignancies	MedImmune LLC	MEDI4736
NCT02013804	Active, not recruiting	I	Advanced Malignancies	MedImmune LLC	–
NCT02271945	Completed	I	Relapsed/Refractory Aggressive B-cell Lymphomas	MedImmune LLC	MEDI-551
**PDR001 (ANTI-PD-1)**					
NCT02678260	Active, not recruiting	I	Advanced Malignancies	Novartis Pharmaceuticals	
NCT02795429	Recruiting	I	Advanced Hepatocellular Carcinoma	Novartis Pharmaceuticals	INC280
NCT02404441	Recruiting	I	Melanoma; NSCLC; TNBC; Anaplastic Thyroid Cancer; Other Solid Tumors	Novartis Pharmaceuticals	
NCT02605967	Recruiting	II	Nasopharyngeal Carcinoma	Novartis Pharmaceuticals	
NCT02947165	Not yet recruiting	I	Breast Cancer; Lung Cancer; Hepatocellular Cancer; Colorectal Cancer; Pancreatic Cancer; Prostate Cancer; Renal Cancer	Novartis Pharmaceuticals	NIS793
NCT02829723	Recruiting	I	Advanced Solid Tumors	Novartis Pharmaceuticals	BLZ945
NCT02936102	Recruiting	I	Advanced Solid Tumors; NSCLC; TNBC; Endometrial Cancer; Anaplastic Thyroid Cancer	Novartis Pharmaceuticals	FAZ053
NCT02988440	Not yet recruiting	I	Hepatocellular Carcinoma	Novartis Pharmaceuticals	Sorafenib
NCT02608268	Recruiting	I	Advanced Malignancies	Novartis Pharmaceuticals	MBG453
NCT02460224	Recruiting	I	Advanced Solid Tumors	Novartis Pharmaceuticals	LAG525
NCT02403193	Recruiting	I	NSCLC	Palobiofarma SL; Novartis; H. Lee Moffitt Cancer Center and Research Institute	PBF-509
NCT02807844	Recruiting	I	TNBC; Pancreatic Carcinoma; Melanoma; Endometrial Carcinoma	Novartis Pharmaceuticals	MCS110
NCT02740270	Recruiting	I	Solid Tumors; Lymphomas	Novartis Pharmaceuticals	GWN323
NCT02967692	Not yet recruiting	III	Melanoma	Novartis Pharmaceuticals	Dabrafenib, Trametinib
NCT02890069	Recruiting	I	Colorectal Cancer; NSCLC(Adenocarcinoma); TNBC	Novartis Pharmaceuticals	LCL161, Everolimus, Panobinostat
NCT02900664	Recruiting	I	Colorectal Cancer; TNBC; NSCLC(Adenocarcinoma)	Novartis Pharmaceuticals	ACZ885, CJM112, TMT212, EGF816
NCT02325739	Recruiting	I	HCC	Novartis Pharmaceuticals	FGF401
NCT02607813	Recruiting	I	NSCLC; Ovarian Cancer; Melanoma; Other Solid Tumors	Novartis Pharmaceuticals; Novartis	LXH254

*Data taken from Clinicaltrials.gov website*.

## PD-1 inhibitors approved by FDA

### Nivolumab

#### In the treatment of melanoma

Metastatic melanoma is one of the most extremely difficult cancers to treat as most conventional cytotoxic and bio-chemotherapy drugs fail to show satisfactory effects. The hope of finding a treatment was improved after the introduction of immune-checkpoint inhibitors (PD-1 and PD-L1 inhibitors) (Pasquali et al., 2017). On December 22, 2014, the FDA approved using Nivolumab (OPDIVO®, Bristol-Myers Squibb), based on clinical trial CA209037), in treating patients suffer from metastatic or unrespectable melanoma and disease that worsens after ipilimumab treatment. Also, Nivolumab is approved for patients who have BRAF V600 mutation-positive or disease progression after BRAF inhibitors (Hazarika et al., 2017). According to trial CA209037, the objective response rate (ORR) in the PDL-1 positive was 43.6%, 20.3% in PDL-1 negative patients, 23.1% in those patients who have BRAF V600 mutation-positive melanoma, and 34% in BRAF wild-type melanoma. Also, Nivolumab showed 30% ORR in those patients who have had a previous response to ipilimumab.

#### In the treatment of head and neck squamous cell carcinoma (HNSCC)

Nivolumab (Opdivo Injection, Bristol-Myers Squibb Company) was approved by the FDA on November 10, 2016, for patients suffering from a recurrent or metastatic form of squamous cell carcinoma of the head and neck (SCCHN) origin, with progression on or after platinum-based chemotherapy. The trial with 361 patients was a multi-center, randomized trial that compared Nivolumab with the investigator's choice (IC) of chemotherapy. The latter included a choice between cetuximab, methotrexate, or docetaxel. The estimated median overall survival was 7.5 months with Nivolumab treatment, compared to 5.1 months for IC.

#### In the treatment of NSCLC

On March 4, 2015, the FDA released a circular for the approval of Opdivo® in the treatment of patients with advanced (metastatic) squamous non-small cell lung cancer (NSCLC) with progression on or after platinum-based chemotherapy. The study was performed in a randomized trial involving 272 participants out of which 135 received treatment with Opdivo and 137 were on docetaxel therapy. After analyzing the results, patients on Opdivo had an overall survival of 3.2 months longer than those on docetaxel.

#### In the treatment of renal cell carcinoma

On November 23, 2015, the FDA approved Opdivo for the treatment of patients with advanced metastatic renal cell carcinoma (RCC) and who have received a certain prior anti-angiogenic cancer therapy. The approval was based on an open-label, randomized clinical study involving 821 patients with advanced RCC. The study was partaken by those whose disease condition had deteriorated after or while on treatment with an anti-angiogenic agent. A certain proportion of patients received Opdivo, while the rest received everolimus (Afinitor). The Opdivo group had an overall survival rate of 25 months compared to 19.6 months in Afinitor group. Moreover, complete or partial tumor shrinkage lasting an average of 23 months was observed in 21.5% patients of the Opdivo treatment arm, compared to 13.7 months in 3.9% of the Afinitor treatment arm.

### Pembrolizumab

#### In the treatment of head and neck squamous cell carcinoma (HNSCC)

On August 5, 2016, the FDA granted approval for pembrolizumab (Keytruda injection, Merck Sharp & Dohme Corp.) for the treatment of patients with recurrent or metastatic head and neck squamous cell carcinoma (HNSCC) with disease progression on or after platinum-containing chemotherapy. The approval was granted based on a multi-center, non-randomized, open-label, multi-cohort study involving 174 patients. With respect to the 28 patients responding to Keytruda treatment, 23 (28%) responded for 6 months or longer.

## PD-1 inhibitors currently under active clinical trials

### In the treatment of renal cell carcinoma

Interventional study type involving an anti-PD-1 drug, BMS-936558 is currently in phase 1 clinical trials (NCT01358721). The study is sponsored by Bristol-Myers Squibb in collaboration with Ono Pharma USA Inc. The trial is the randomized type with a parallel assignment with intravenous infusion of the solution at doses 0.3, 2, and 10 mg/kg, every 3 weeks.

### In the treatment of NSCLC

Interventional study type involving Pembrolizumab (MK-3475) is also in phase 1 clinical trials (NCT01295827). The study is sponsored by Merck Sharp & Dohme Corp. and has randomized, parallel assignment, interventional study design with the drug dosing at 1, 3, and 10 mg/kg.

### In treatment of HNSCC

This study is being done to examine the safety, tolerability, and anticancer activity of pembrolizumab (MK-3475) in patients with advanced triple negative breast cancer (TNBC) (Cohort A), advanced head and neck cancer (Cohorts B and B2), advanced urothelial cancer (Cohort C), or advanced gastric cancer (Cohort D). The study is currently in Phase 1 clinical trials (NCT018488344), sponsored by Merck Sharp & Dohme Corp. and is an interventional, non-randomized, parallel assignment, open-label study design.

## PD-L1 currently under active clinical trials

BMS-936559, Avelumab, MEDI4736, and, Atezolizumab are the anti-PD-L1 antibodies that are being studied for various cancers and tumors including melanoma, multiple myeloma, leukemia, lymphoma, glioblastoma as well as gastric, renal cell, bladder, colorectal, hepatocellular, cutaneous, breast and NSCLC (Non-Small Cell Lung Cancer) cancers, (CRC), castration-resistant prostate cancer (CRPC), renal cell carcinoma (RCC), head and neck squamous cell cancer (HNSCC). They suppress the T-cells and increase the production of the regulatory T-cells, thus reducing the auto-immunity and improving the tolerance (Francisco et al., 2010) of some of these ligands promote the complement-dependent cytotoxicity.BMS-936559 is being studied by Bristol Myers Squibb with one study recruiting for Phase I/II trials. Avelumab is being developed by Merck Sharp Dohme with about 23 trials recruiting for further studies some of which are at the Phase 3 level (Li et al., 2016) that include combination therapies. MEDI4736 is another promising PD-L1 ligand that has trials recruiting for Phase 3 level trials mostly for combination therapy. Atezolizumab is an FDA approved the drug for urothelial carcinoma which also has some Phase 3 trials going on for other types of cancer as shown in Table 1. It is being marketed under the trade name Tecentriq® by Genentech Inc. So far, only one PD ligand has been approved and is being used successfully both in single-drug therapies and in combination therapies. Thus, in all, there are more than 300 clinical trials registered for these drugs for various cancer states that are at different phases.

## PD-L1 inhibitors approved by FDA

### Atezolizumab

#### In the treatment of NSCLC

On October 18th, 2016, the FDA approved using Atezolizumab for treating patients with metastatic non-small lung cell lung cancer (NSCLC) in which their disease worsens during or after platinum-containing chemotherapy. The approval of Atezolizumab relied on two international, randomized, open label clinical trial that had 1,137 patients with NSCLC. These studies showed the efficacy and safety of Atezolizumab in those patients. Atezolizumab showed improvements in the survival rate in comparison to docetaxel in both clinical trials by 4.2 and 2.9 months. Patients who have epidermal growth factor receptor (EGFR) and anaplastic lymphoma kinase tumor (ALKT) can start Atezolizumab treatment in case of failure of the appropriate therapies.

#### In the treatment of urothelial carcinoma

On May 18, 2016, the FDA approved using Atezolizumab for treating patients who have advanced or metastatic urothelial carcinoma in which their diseases worsen during or after using platinum-containing chemotherapy. Atezolizumab was approved based on a multi-center single-arm trial that had 310 patients. It was found that Atezolizumab showed good activity as immune checkpoint inhibitors. It was noted that as the expression of PD-L1 increases, the response increases (Rosenberg et al., 2016).

## PD-1 inhibitors for combination therapy

Targeting T-cells regulatory proteins, such as CTLA-4 and PD-1 by checkpoint blocking antibodies has been strengthened the area of cancer immunotherapy. The FDA approval of CTLA-4 antibody inhibitor (ipilimumab) and PD-1 inhibitors (Pembrolizumab, Nivolumab) have diversified the clinical activity toward wide variety solid tumor including lung cancer, renal cell cancer, and ovarian cancer. Based on clinical data these monotherapeutics have been demonstrated to be a successful immunotherapy regimen. Considering the safety and better clinical activity of monotherapy the field is moving toward the direction of discovering novel combination therapies (Pardoll, 2012). Various anti-cancer agents including other check point inhibitors, kinase inhibitors, chemotherapeutics, and targeting agents are used in combination with PD-1 antibody inhibitors (Callahan et al., 2014).

### Combination of PD-1 and other checkpoint inhibitors

The anti-CTLA-4 antibody, ipilimumab, has shown durable anti-tumor activities and prolonged survival in participants with advanced melanoma, resulting in its Food and Drug Administration (FDA) approval in 2011 (Hodi et al., 2010). Although, this approach serves as proof of concept for the possible activity of a checkpoint blockade, ipilimumab, perhaps because of its function in the priming phase of the immune response, it also appears to activate immunity against many types of normal tissues causing severe immune-related adverse events (irAEs) in a substantial minority of patients. Therefore, more recent research has concentrated on the restoration of anti-tumor immunity selectively within the tumor microenvironment through the utilization of humanized antibodies that inhibit the PD-1–PD-L1 signaling. The combination of ipilimumab and Nivolumab are undergoing human trials for clear-cell metastatic renal cell carcinoma (CCRCC), advance melanoma, and non-small cell lung carcinoma. A phase 1 (NCT01472081) study in CCRCC showed promising safety and tolerability up to 100 days after the last dosing (Callahan et al., 2014). A recent phase III trial (NCT01844505) reported the treatment of Nivolumab monotherapy or a combination of Nivolumab with ipilimumab for unresectable or metastatic melanoma. The combination therapies of PD-1 or PDL-1 with other checkpoint inhibitors are in clinical trials, such as (i) a phase I study (NCT01968109) of “nivolumab + BMS-936558” for targeting PD1/LAG3 in advance solid tumor; (ii) a phase II study (NCT02845323) of “nivolumab + urelumab” for targeting PD1/4-1BB in B-cell lymphoma; and (iii) a phase II study (NCT02543645) of “atezolizumab + varlilumab” for targeting PDL1/CD27 in RCC (Morrissey et al., 2016). The overall data of combination immunotherapy offer promising future cancer treatments.

### Combination of PD-1 and kinases inhibitors

Mitogen-activated protein kinase (MAPK) is an effective regulator of BRAF mutation, which is responsible for the metastasis of cutaneous melanoma (Wilmott et al., 2012). In the clinic, BRAF inhibition has improved metastasis of melanoma, and during treatment, tumors exhibited a significant increase in CD8^+^ T cells, which is linked to the down regulation of vascular endothelial growth factor (VEGF) and IL-6, IL-1α cytokines. The mutation of BRAF has also increased the expression of the PD-1 and PDL-1 molecule and induces potential drug resistance with the involvement of tumor stromal cells. The combination of PD-1 immunotherapy with a BRAF inhibitor resulted in synergistic anti-tumor response and prominent tumor growth inhibition (Azijli et al., 2014).

There is proof that VEGF suppresses dendritic cell (DC) functions; thus, restoring DC and T-cell activity could improve immune response and anti-tumor activity. A phase I clinical study (NCT01454102) of VEGF inhibitors (bevacizumab) combined with Nivolumab is ongoing for stage III NSCLC patients and unresectable stage III or IV melanoma (McCabe and Creasy, 2014). Another phase I/II study (NCT02130466) is recruiting melanoma and other solid tumor patients using BRAF inhibitor and MEK inhibitor with a combination of Pembrolizumab (Morrissey et al., 2016).

### Combination of PD-1 and chemotherapeutics

PD-1 inhibitors are being treated in combination with antineoplastic agents, such as cisplatin, to achieve a long-lasting synergistic anti-cancer effect. For example, (i) a phase 3 study (NCT02494583) is recruiting gastric adenocarcinoma patients for combination of pembrolizumab either with cisplatin or capecitabine or 5-fluorouracil, and (ii) another phase I/II study (NCT02077959) for refractory multiple myeloma has been pursuing with pidilizumab (PD-1 inhibitor antibody) and lenalidomide (Morrissey et al., 2016).

Recent trends in cancer treatment are moving toward combination immunotherapy, but its success depends on addressing the challenges of choosing the right drug combination, optimizing the dose and schedule of the combination regimen, and managing toxicities and side effects.

## Safety concerns related to PD-1 and PD-L1 immunotherapy

Once the immune checkpoints have been blocked, the equilibrium between the autoimmunity and immune tolerance will be affected. The term immune-meditated adverse reactions (IMARs) is a term coined to describe the side effects of new immunotherapy. The most frequent events of any adverse effect that happened to patients with melanoma were fatigue (32%), rash (23%), and, skin disorders (36%), GI events (18%), endocrinopathies (13%), and diarrhea (18%). In addition to the above immune-mediated complications, pneumonitis, colitis, hepatitis, endocrinopathies, encephalitis were also associated with the therapy. Some patients also suffered from nephritis and renal dysfunction, acute kidney injury, pleural effusion, and hypercalcemia. Side effects to the patients undergoing Nivolumab therapy required the use of corticosteroids and had no clear alternative etiology. In general, toxicities with anti-PD-1/PD-L1 mAbs appear to be less common and less severe when compared with anti-CTLA-4 mAbs. Use of anti-PD-1 and PD-L1 therapy to patient results immune-related adverse events (irAE) that is associated with diarrhea, colitis, pancreatitis and neurologic adverse effects, hematologic adverse effects, and Pneumonitis. The longer course treatment with anti-PD-1/PD-L1 therapy causes severe immune responses. Abnormalities of hepatic enzymes (AST and ALT) in serum level have been reported for anti-PD-1 therapy (Postow et al., 2015). Several questions remain unanswered regarding the optimal dose and schedule of PD-1/PD-L1 checkpoint blockers. Multiple studies have reported that 1 mg/kg PD-1 inhibitors will be the optimal dose and increase of dose does not enhance efficacy.

## Conclusion and future prospective

Cancer immune checkpoint inhibitors including PD-1/PD-L1 have made a remarkable journey from the bench to the bedside over the past few years. The development and antibody targeting of immune checkpoint mechanisms such as the PD-1/PD-L1 pathway has led to a clinically significant antitumor response. Rapid improvement is being demonstrated in the treatment of various tumor types resulting in the first FDA approval for this newly discovered agent in patients with advanced melanoma based on meeting high safety and efficacy standards. Currently, multiple studies are estimated to establish the value of PD-1 pathway inhibitors in other cancer types as well as in combination with previously FDA approved therapeutics. However, considering that only some patients will be able to take advantage of such therapy, and the likely cost of such treatments, it is fair to expedite biomarker development to focus therapy on patients who will be most likely benefit and be the chosen candidates for clinical trial enrichment during the FDA approval process of these novel agents. The immunotherapy success story in the management of advanced cancers will lead to a more efficient use of these agents at an earlier stage of the disease in single or in combination with other chemo or immunotherapeutic medications or other modalities, such as surgery or radiation, which can significantly increase overall survival or cure rates. A better understanding of the tumor immune microenvironment with other proposed hallmarks of cancer will advance the knowledge and research in cancer development and progression and eventually result in impactful advances in cancer therapy and management. Various types of nanoparticles have been utilized for cancer drug delivery (Sau et al., 2014; Alsaab et al., 2017; Gawde et al., 2017; Luong et al., 2017; Sahu et al., 2017) and current research outcomes are coming out to target the immune check point inhibitors for cancer therapy (Wang et al., 2016). Despite clinical success of anti-PD-1/PD-L1 immunotherapy, some fraction of patients fails to respond to these therapies (Philips and Atkins, 2014; Tumeh et al., 2014). The reasons of unresponsive to immunotherapy are attributed to multiple factors, such as (i) insufficient infiltration of activated CD8+ T cells into the non-inflamed types of tumor microenvironment that retards anti-PD-1 targetability (Spranger et al., 2016), (ii) variable population of CD8+ T cells from edge to core of the tumors due to tumor heterogeneity, hypoxia, and variability in mutations of specific oncogene pathways (Gajewski et al., 2013). The proposed strategy for improving anti-PD-1/PD-L-1 therapy involves combining PD-1 inhibitor with intratumoral delivery plasmid DNA encoding an immune stimulating cytokine, such as interleukin-12 (or IL-12) (Pisetsky, 1996). There is need of highly sensitive assay for identification biomarker expression of a patient population to determine feasibility of PD-1/PD-L1 therapy.

## Author contributions

HA, SS, RA, KT, KB, and AI summarized the literature and drafted the manuscript. HA, SS, SK, and AI revised and edited the manuscript. AI supervised the work. AI and SS initiated, finalized, and submitted the manuscript.

### Conflict of interest statement

The authors declare that the research was conducted in the absence of any commercial or financial relationships that could be construed as a potential conflict of interest.
